# Poly‐L‐Lactic Acid Combined With CO_2_ Fractional Laser for the Treatment of Acne Scars

**DOI:** 10.1111/jocd.70271

**Published:** 2025-06-06

**Authors:** Chenxi Zhou, Weiliang Chen, Liyuan Zhang, Xiao Luo, Lin Zhu, Erting Zhang, Kai Li, Xiaolei Qin

**Affiliations:** ^1^ DEXI Clinc, DE YI Skin Xi'an Shanxi China; ^2^ DEYUE Clinc, DE YI Skin Shenzhen China; ^3^ Peking University Shenzhen Hospital Shenzhen China

**Keywords:** acne scar, CO_2_ fractional laser, PLLA, poly‐L‐lactic acid

## Abstract

**Background:**

The treatment of acne scars has always been challenging. In the past, its gold standard treatment was carbon dioxide (CO_2_) fractional laser, but its efficacy rate was only 51%–70%. The use of poly‐L‐lactic acid (PLLA) has provided a new treatment strategy. In this study, a novel PLLA material was used in combination with a CO_2_ fractional laser to treat acne scars, and its safety and effectiveness were evaluated.

**Aims:**

To determine the safety and effectiveness of PLLA (Loviselle) combined with a CO_2_ fractional laser in the treatment of acne scars.

**Methods:**

In this study, 60 patients were randomly divided into two groups, with 30 patients in each group. Group A received only CO_2_ fractional laser treatment, whereas Group B received injections of PLLA 1, 3, and 5 months after CO_2_ fractional laser treatment. Both groups were followed up for 6 months after the CO_2_ fractional laser treatment.

**Results:**

There were statistically significant differences in the Quantitative Global Scarring Grading System (GSS) scores between the two groups before and after treatment. Group B had lower GSS scores than Group A. In the treatment of different types of acne scars using the Acne Scar Rating Scale, the improvement in rolling scars was greater than that in boxcar scars, and both were greater than those in ice‐pick scars. Whether evaluated by independent physicians' assessment of treatment improvement or patients' satisfaction questionnaire, the combined treatment group showed better results than the single treatment group, and there were statistically significant differences between the two groups. Adverse reactions included transient redness, bruising, and pigmentation.

**Conclusions:**

The combination of CO_2_ fractional laser and PLLA can safely and effectively treat acne scars, especially rolling and boxcar depressed scars.

## 1 Introduction

Acne vulgaris, a prevalent inflammatory skin condition, often leads to lasting sequelae in up to 80.2% of patients, with approximately 43% developing acne scars [1]. The presence of these scars can significantly affect a patient's self‐esteem and mental well‐being. Despite advances in treatment modalities, management of acne scars remains a clinical challenge. Conventional treatments range from multiple sessions of chemical peeling, laser, and radiofrequency applications to injections of various materials, such as hyaluronic acid, poly L‐lactic acid (PLLA), and calcium hydroxylapatite [2].

Among the various options for treating atrophic acne scars, carbon dioxide (CO_2_) fractional laser therapy is commonly used, regardless of the scar type (rolling, boxcar, or ice‐pick). However, the efficacy of CO_2_ fractional laser therapy is limited, with reported success rates ranging from 51% to 70% [2]. Furthermore, this treatment often requires multiple sessions and is accompanied by postoperative adverse reactions, such as erythema and pigmentation [3].

For a long time, injection therapy has emerged as a safe and effective alternative for acne scar management [4]. Specifically, PLLA injection therapy offers a relatively short recovery period and rapid healing of acne scars. However, the incidence of adverse reactions following treatment, including the development of small, invisible nodules after 1 year, can be as high as 36.4%, posing a challenge to management [5]. Although Sculptra, a PLLA injection material from the United States, has been widely used in the past, the material employed in this study, Loviselle, is a novel material, just 3 years on the market. Loviselle features a diameter of 25–50 μm, a smooth spherical shape, and uniform metabolism over a 2‐year period, thereby reducing the likelihood of nodule formation [6]. But the reporting of the treatments, which involves relatively simple trial designs and a relatively short follow‐up period, presents a challenge. Therefore, the clinical treatment effects, adverse events, and patient satisfaction of Loviselle in China should be objectively assessed [7].

This study aimed to evaluate the safety and efficacy of this innovative PLLA material (Loviselle) in combination with CO_2_ fractional laser therapy for the treatment of acne scars. This represents the first clinical observation of Loviselle's application in acne scar management since its approval by the China Food and Drug Administration 3 years ago.

## 2 Materials and Methods

### 2.1 Inclusion and Exclusion Criteria for Participants

The inclusion criteria were as follows: (1) age > 18 years, regardless of sex; (2) clinical diagnosis of mild‐to‐moderate facial acne scars; and (3) complete medical records and compliance with the study protocol.

The exclusion criteria were as follows: (1) pregnant or potentially pregnant women; (2) patients with concurrent systemic diseases, such as cardiovascular, cerebrovascular, liver, or kidney disease, epilepsy, diabetes, or lupus erythematosus; (3) photosensitive patients; (4) patients with a history of mental illness or psychological disorders; (5) patients with skin lesions with local ulceration, active bacterial, viral, or fungal infections, or suspected skin cancer in the treatment area; moderate to severe acne; (6) patients with concurrent active vitiligo or psoriasis; (7) patients who had used hydroxy acids or glycolic acid locally within 3 months prior to the study or had undergone intense pulsed light therapy, fractional laser therapy, radiofrequency microneedling, or other facial treatments within 3 months prior to the study; (8) patients who had undergone filling injection treatment within the past 6 months; and (9) patients who had hypertrophic and keloid tendency. Throughout the study period, all participants were instructed to refrain from any energy‐based, whole‐body, or local treatments.

### 2.2 Testing Methods

Prior to treatment, all patients underwent a comprehensive facial assessment and skin cleansing. Standardized facial photographs were captured using a Think view skin analyzer (Taiwan) and a digital camera (Canon Powershot S95) under controlled lighting conditions from the front, left side (45° and 90°), and right side (45° and 90°) for documentation and follow‐up comparisons. Written informed consent was obtained from all the participants. Photos were taken before applying local anesthesia.

To achieve superficial facial anesthesia, the treatment area was coated with a compound lidocaine cream (2.5% lidocaine and 2.5% prilocaine, 50 mg; Beijing Ziguang Pharmaceutical Co. Ltd., China) and covered with plastic wrap for 30 min.

**FIGURE 1:**
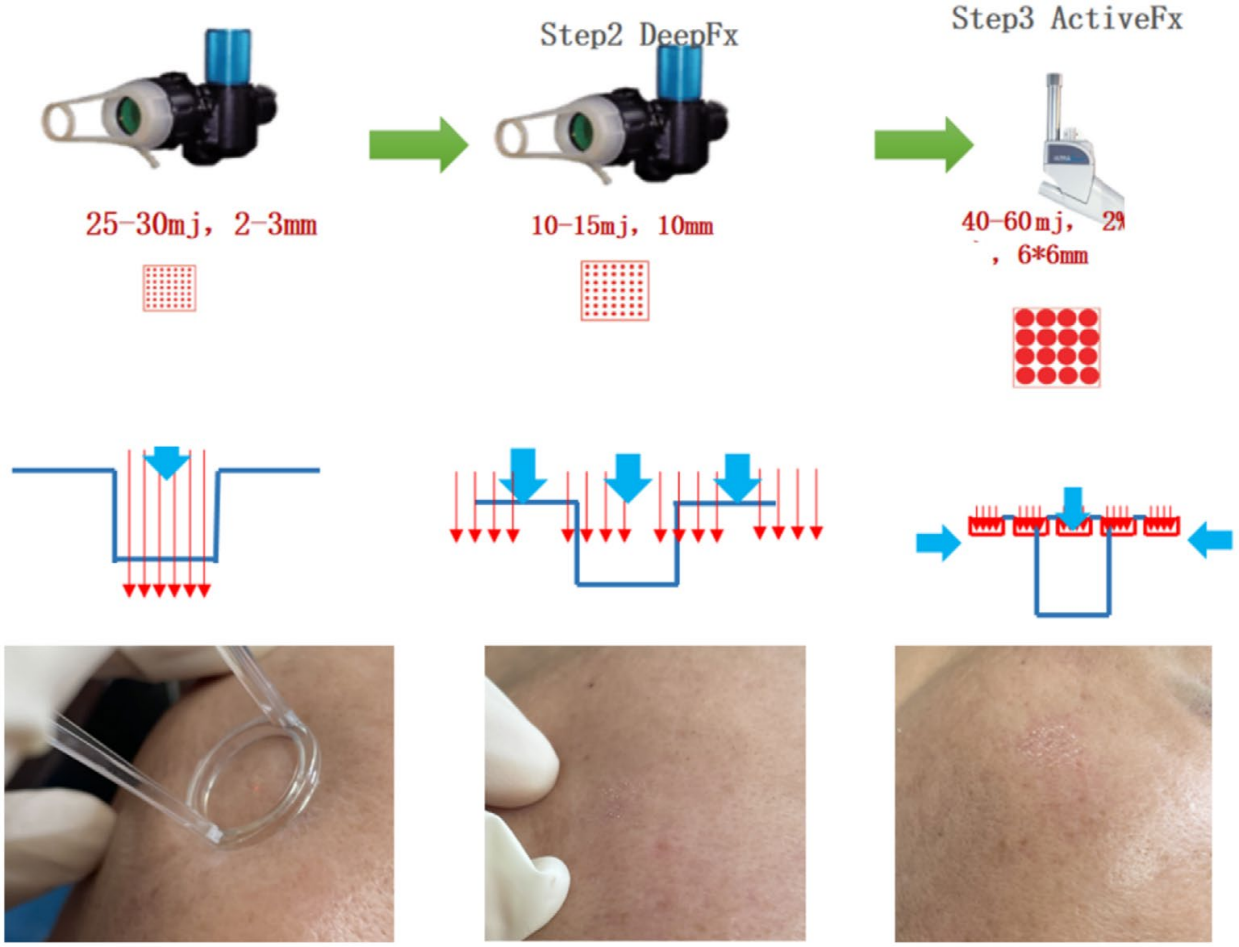
Fractional laser treatment for acne scars.

This study was designed as a randomized, prospective clinical trial. Participants with facial acne scarring were recruited through online leaflet distribution. Sixty patients were randomly divided into two groups: Groups A and B. Group A received only CO_2_ fractional laser treatment using an UltraPulse system (UP, USA). The treatment parameters were as follows: for the DeepFx handpiece, an energy level of 25–30 mj and a spot size of 2–3 mm were initially applied to the deepest part of the scar, followed by covering the entire scar area with 10–15 mj of energy and a spot size of 10 mm × 10 mm. Depending on the scar condition, the ActiveFx handpiece was used with a density of 2%, an energy level of 40–60 mj, and the depth was about 70–90 μm. If the scar was sharp, high energy was used. The spot size was 6 mm × 6 mm. The pulse duration was 90–290 μs (Figure 1). The laser treatment was undergone a single time. During and after laser treatment, the patients were instructed to use an air‐cooling device (Zimmer cryo6) to mitigate erythema, swelling, and discomfort.

The patients were advised to avoid photosensitive foods and limit sun exposure for 1 month following treatment, utilizing physical sun protection measures when outdoors. Follow‐up assessments were scheduled 6 months after the initial laser treatment.

Group B received the same CO_2_ fractional laser treatment as Group A followed by PLLA (Loviselle) filling injections 1, 3, and 5 months after the laser treatment.

After undergoing CO_2_ fractional laser treatment, Group B received PLLA (Loviselle; Changchun Shengboma Biomedical Company, China) injections at 1, 3, and 5 months posttreatment. The preparation method involved reconstituting 340 mg of freeze‐dried powder (containing 150 mg of polylactic acid) in 4–5 mL of normal saline and shaking for 5 min to achieve a uniform mixture.

**FIGURE 2:**
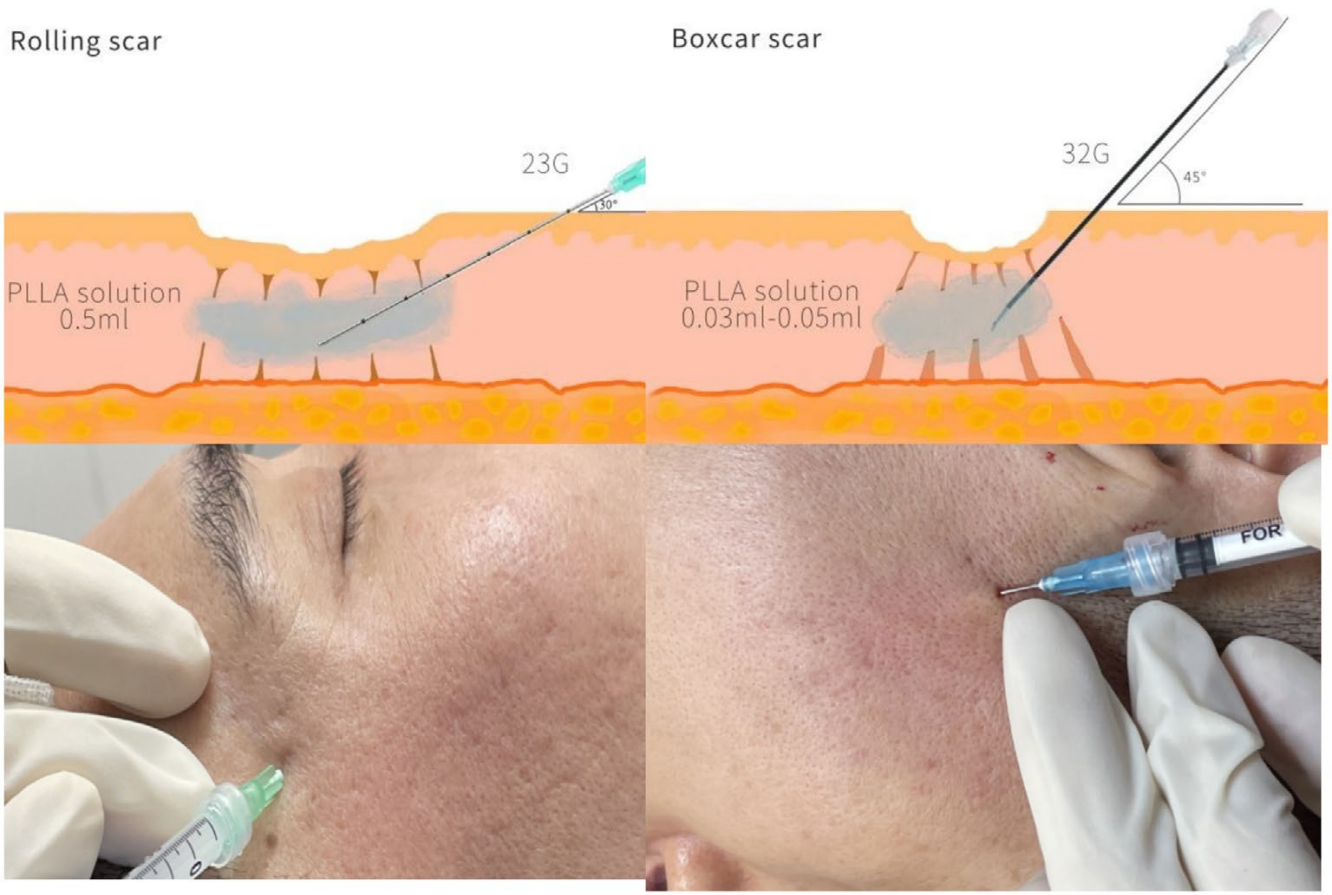
Poly‐L‐lactic acid injection for acne scars.

The injection technique varied according to the type of acne scarring (Figure 2). For boxcar‐type depressed scars, a 32G, 4‐mm sterile needle was used to inject the PLLA solution at 45° into the side of the scar, aiming to level the depressed lesion skin with the surrounding normal skin. Typically, no more than 0.3 mL of PLLA solution (containing 15 mg of polylactic acid) was administered per square centimeter of scar area.

For rolling‐type depressed scars, a 23G blunt needle was used, and the solution was injected linearly along the rolling scar. The dose administered per injection channel was < 0.5 mL. If the skin was not flat, the physician gently massaged the treated area to achieve optimal flattening.

A unique injection technique involved initially administering 3–4 mL of the PLLA solution while the patient was in the supine position. Subsequently, the remaining 1–2 mL was injected while the patient was in a sitting position, considering the potential variation in scar morphology between these two positions. The patients were instructed to avoid contact with water for 24 h after each injection.

### 2.3 Efficacy Formulation Criteria

#### 2.3.1 Severity of Acne Scars

The severity of acne scars was quantitatively evaluated by two independent physicians using the Quantitative Global Scarring Grading System (GSS). This system assigns a composite score ranging from 0 to 84 based on the overall severity of acne scars before and after treatment [8]. Physicians' scores were averaged to obtain a representative severity index for each patient.

To gain further insights into the effectiveness of treatment for different scar types, the Acne Scar Rating Scale (ASRS) was used by the same independent physicians to assess patients in the combined treatment group. The ASRS is a 4‐point scale, which is classified as follows: 1 = minimal, 2 = mild, 3 = moderate, and 4 = severe [9]. The average ASRS score was recorded, and the difference between the preoperative and postoperative ASRS scores was calculated.

#### 2.3.2 Evaluation of the Degree of Improvement

To assess the degree of improvement in acne scars following treatment, two independent physicians utilized a 4‐point global assessment score (GAS). The GAS score ranged from 0 to 4, with 0 representing no improvement or worsening of the scars, 1 indicating slight improvement (≤ 25%), 2 representing moderate improvement (26%–50%), 3 indicating substantial improvement (51%–75%), and 4 indicating significant improvement (76%–100%). The physician evaluations were averaged to obtain a comprehensive score for each patient.

All the patients were invited to participate in a satisfaction survey after treatment completion. This survey aimed to capture the patients' overall evaluation of the improvement in their acne scars. The patients were instructed to rate their satisfaction on a scale of 0–4, where 0 signified very dissatisfied; 1, neutral; 2, average; 3, satisfied; and 4, very satisfied. Patient satisfaction scores were compiled and analyzed to provide insights into the treatment's perceived effectiveness from the patients' perspective.

#### 2.3.3 Adverse Events

A Numeric Rating Scale (NRS) was used to comprehensively evaluate adverse events associated with the treatment. The scale ranges from 0 to 10, with 0 indicating no pain and 10 indicating the worst imaginable pain. At each follow‐up visit, all patients were instructed to rate the severity of treatment‐related pain using this scale. Additionally, any adverse events, including erythema, swelling, bruising, pigmentation changes, nodule formation, or other unexpected reactions, were meticulously recorded and evaluated during the final follow‐up assessment.

Prior to initiating treatment, the patients were thoroughly informed about potential adverse events and reminded to seek prompt medical consultation or assistance at nearby hospitals in cases of severe or urgent symptoms during the follow‐up period. This precautionary measure aimed to ensure the safety and well‐being of all the participants throughout the study.

### 2.4 Statistical Analyses

Statistical analyses were performed using the SPSS software (version 26.0; IBM Corp., Armonk, NY, USA). Descriptive statistics are presented as means ± standard deviations for normally distributed variables, medians (lower quartiles, upper quartiles) for nonnormally distributed variables, and percentages (%). Categorical variables were analyzed using the chi‐squared or Fisher's exact test depending on the expected cell frequencies. Normally distributed numerical data were compared between groups using independent *t*‐tests, whereas for nonnormally distributed numerical data, the Mann–Whitney U test was employed to determine the statistical significance of differences between the two treatment groups. The Wilcoxon signed‐rank test was used to compare the pre‐ and posttreatment scores within each group. For comparisons involving three or more groups, one‐way analysis of variance with Bonferroni correction for multiple comparisons was applied. All statistical tests were two‐tailed, and a *p* value < 0.05 was considered statistically significant.

## 3 Results

### 3.1 General Information

The study cohort comprised 50 females (83.3%) and 10 males (16.7%), with Fitzpatrick skin types ranging from II to IV. The participants were aged between 18 and 42 years, with a mean age of 28.88 ± 3.93 years. The duration of acne scarring ranged from 2 to 20 years, averaging 7.78 ± 3.62 years. Prior to treatment, the overall severity of acne scars was assessed using the GSS, with scores ranging from 10 to 58 and a median of 26.00 (19.00–35.00). As shown in Table 1, no statistically significant differences were observed in the baseline clinical characteristics between the two treatment groups (*p* > 0.05), ensuring comparability at the beginning of the study.

**TABLE 1:** Baseline clinical data of patients.

		N (%) or mean ± SD or median (lower quartile, upper quartile)		t or Z	χ^2^	*p*
		Group A	Group B			
Sex	Male	6 (20.0)	4 (13.3)	—	0.480	0.488
	Female	24 (80.0)	26 (86.7)			
Fitzpatrick skin type	II	5 (16.7)	5 (16.7)	—	0.439	0.803
	III	20 (66.7)	18 (60.0)			
	IV	5 (16.7)	7 (23.3)			
Age (years)		28.03 ± 4.11	29.73 ± 3.62	1.701	—	0.094
Duration (years)		7.47 ± 3.99	8.10 ± 3.25	0.674	—	0.503
GSS (points)		26.00 (15.75–39.25)	25.00 (20.75, 28.00)	0.027	—	0.970

Abbreviations: GSS, Quantitative global scarring grading system; SD, Standard deviation.

### 3.2 Severity of Acne Scars

**FIGURE 3:**
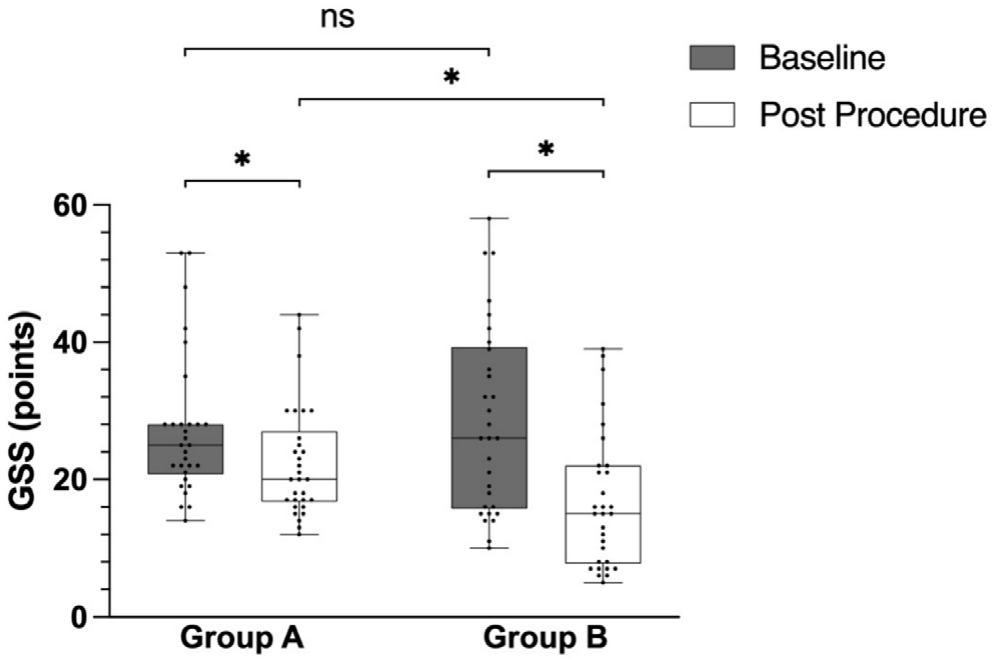
Changes in the quantitative global scarring grading system (GSS) of the two groups. ns: *P* = 0.970, *p < 0.05.

Intragroup comparisons revealed statistically significant reductions in the severity of acne scars following treatment in both groups, as assessed by the GSS (Figure 3). Specifically, Group A (CO_2_ fractional laser alone) exhibited a significant difference before and after treatment (Z = 4.084, *p* < 0.001), whereas Group B (combined CO_2_ fractional laser and PLLA injection) demonstrated a significant reduction (Z = 4.789, *p* < 0.001).

Intergroup comparisons further highlighted the efficacy of the combined treatment approach. Group B achieved a lower median GSS after treatment compared with Group A (15.00 [7.75–22.00] vs. 20.00 [16.75–27.00], respectively; Z = 2.709; *p* = 0.007) (Figure 3). This finding suggests that the combination of the CO_2_ fractional laser and PLLA injection results in a more pronounced improvement in acne scar severity compared with CO_2_ fractional laser alone.

To delve deeper into the treatment efficacy for different types of depressed acne scars, we analyzed the ASRS scores. All three scar types (ice‐pick, rolling, and boxcar) exhibited statistically significant improvements in ASRS scores after treatment (*p* < 0.05) (Table 2). However, pairwise comparisons using Bonferroni correction revealed differences in the degree of improvement among the scar types. Specifically, the combined treatment led to a greater improvement in rolling scars compared with boxcar scars (*p* < 0.05), and both rolling and boxcar scars showed greater improvement than ice‐pick scars (*p* < 0.05). These findings indicate that the combined treatment approach is particularly effective for rolling and boxcar scars, whereas ice‐pick scars tend to respond less favorably.

**TABLE 2:** Comparison of improvement in different acne scar types based on combination therapy.

	Cases	Acne scar rating scale (ASRS, points)		
		Preoperative	Postoperative	Difference
Ice‐pick	30	2.02 ± 0.18	1.70 ± 0.16^1^	0.32 ± 0.07
Boxcar	30	2.30 ± 0.16	1.52 ± 0.11^1^	0.78 ± 0.11^a^
Rolling	29	2.41 ± 0.14	1.10 ± 0.09^1^	1.31 ± 0.11^a^, ^b^
*F*		1.602	7.236	26.334
*p*		0.207	0.002	< 0.001

^1^ versus preoperative, *p* < 0.05.

^a^ versus ice‐pick, *p* < 0.05.

^b^ versus boxcar, *p* < 0.05.

### 3.3 Evaluation of Treatment Improvement

**FIGURE 4:**
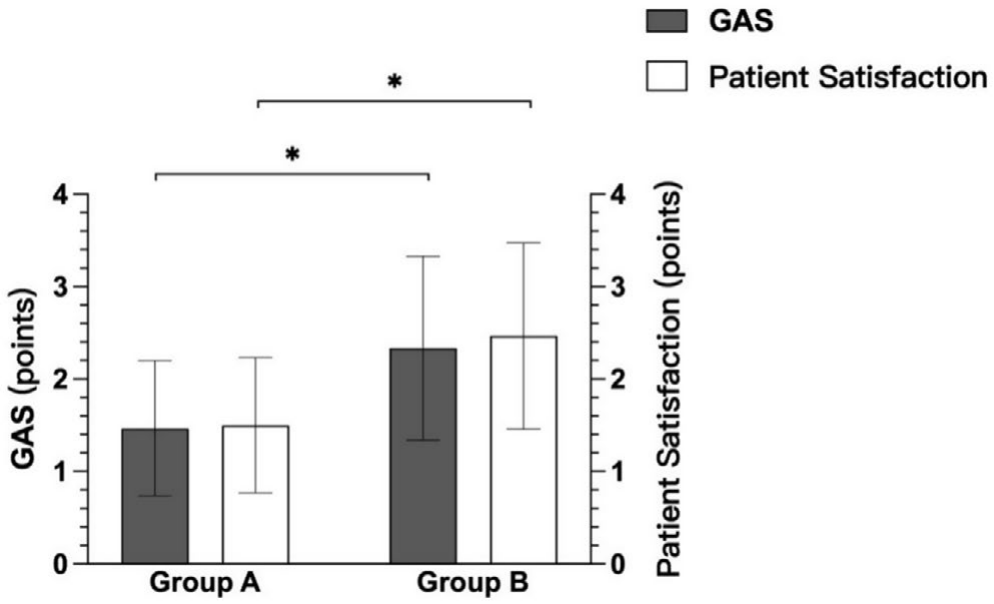
Changes in the global assessment score (GAS) and patient satisfaction of the two groups. *p < 0.001.

The assessment of treatment improvement, both from the perspective of independent physicians and patients, revealed superior outcomes in the combined treatment group compared with the single treatment group. The independent physicians' GAS for treatment improvement was significantly higher in Group B (combined treatment) than in Group A (single CO2 fractional laser treatment) (1.47 ± 0.73 vs. 2.33 ± 0.99, t = 3.848, *p* < 0.001) (Figure 4). Similarly, patient satisfaction, as captured by the satisfaction questionnaire, was also significantly greater in Group B compared with Group A (1.50 ± 0.73 vs. 2.47 ± 1.01, t = 4.252, *p* < 0.001).

However, notably, in Group A, two patients exhibited an increase in their GSS following treatment. Both patients scored 0 on the GAS for treatment improvement and reported dissatisfaction with the satisfaction survey. This finding underscores the variability in treatment responses and highlights the need for individualized treatment plans and follow‐up assessments.

### 3.4 Adverse Reactions

The assessment of adverse reactions revealed minimal and transient side effects in both treatment groups. Pain levels, measured using the NRS, ranged from 4 to 7, indicating moderate discomfort during and after the treatment. Although the mean NRS score was slightly higher in Group B compared with Group A (5.43 ± 0.97 vs. 5.83 ± 0.99), this difference was not statistically significant (t = 1.583, *p* = 0.119).

**FIGURE 5:**
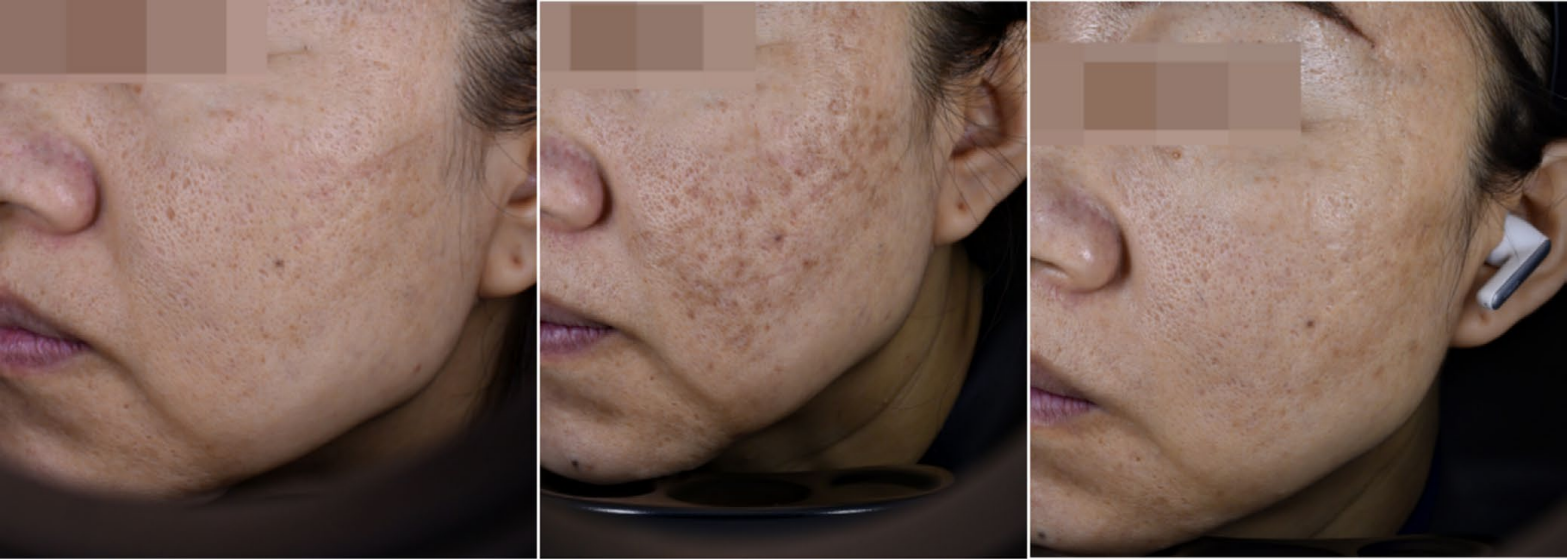
Before UP treatment for acne scar. One month after UP treatment for acne scar. Six months after UP treatment for acne scar.

In Group A, four (13.3%) patients reported posttreatment erythema, which lasted for 1–2 months. Additionally, two (6.7%) patients experienced postinflammatory hyperpigmentation, which resolved spontaneously within 3–6 months. One of the patients who had melasma, with hyperpigmentation lasting for 6 months (Figure 5), After taking tranexamic acid tablets for 3 months, the hyperpigmentation disappeared. In contrast, Group B exhibited more frequent but shorter lived erythema and bruising. Specifically, 10 (33.3%) patients developed posttreatment erythema and bruising, but these adverse reactions were transient, lasting only 7 days, and resolved without the need for medical intervention.

No skin nodules, hypertrophic scars, or other severe adverse reactions were observed in either treatment group during follow‐up. This finding suggests that both treatments are generally well‐tolerated, with a minimal risk of long‐term complications.

## 4 Discussion

Ablative fractional laser is still considered the primary treatment of atrophic acne scars; however, its effectiveness ranges from 51% to 70% [2]. In the past years, PLLA has been commonly used to treat atrophic acne scars. The main principles behind how PLLA fillers improve acne scars include the following: [10] (1) initiation of a regenerative signal for collagen synthesis by lactic acid, which is achieved by increasing the levels of tumor growth factor‐β; (2) upregulation of the tissue inhibitor of metalloproteinase 1 signaling pathway, which increases the activity of metalloproteinase inhibitors; (3) stimulation of the activity of prolyl hydroxylase by lactic acid, increasing hydroxyproline levels; and (4) metabolism of lactic acid providing energy for collagen synthesis. Historically, PLLA treatments have included radiofrequency microneedling [11], blunt cannula injections [5], and needle‐free delivery [12]. However, there are currently no studies comparing the effectiveness of combined CO2 fractional laser and PLLA therapy, and there are no reports on the use of different PLLA treatment modalities for various types of acne scars.

**FIGURE 6:**
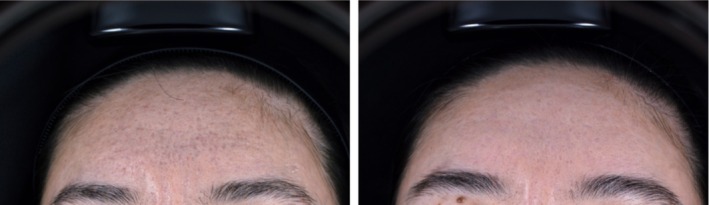
The efficacy of poly‐L‐lactic acid combined with carbon dioxide fractional laser treatment on the forehead.

**FIGURE 7:**
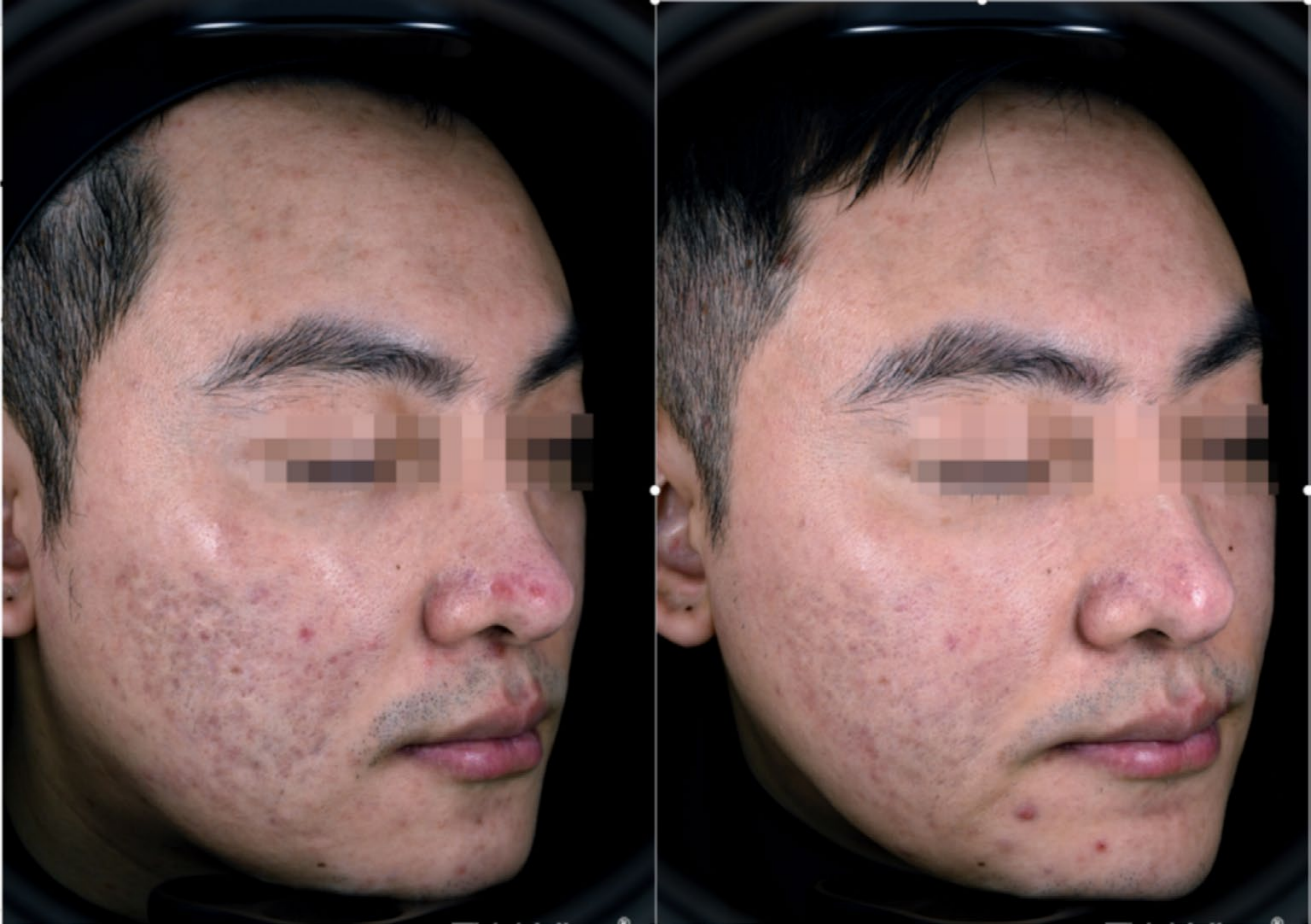
The efficacy of poly‐L‐lactic acid combined with carbon dioxide fractional laser treatment on the cheeks.

In this study, the GSS scores decreased in both treatment groups, with the combined therapy proving superior in the intergroup comparison. However, two patients in Group A experienced an increase in GSS, which is hypothesized to be related to acne recurrence after CO_2_ fractional laser treatment. In terms of the ASRS scores, we observed that rolling acne scars responded best to the combined PLLA therapy, followed by boxcar scars, whereas ice‐pick scars showed less favorable results. This difference in efficacy may be attributed to the distinct shapes of acne scars (Figures 6 and 7).

Based on the classification and morphological characteristics of acne scars [13], ice‐pick scars are narrow, deep, and punctate, making it difficult for PLLA to act uniformly in narrow and deep skin tunnels. In contrast, boxcar scars have a broader base and vary in depth, allowing the medication to disperse evenly at the bottom during sharp needle injections, resulting in a leveling effect. There is a paper indicating that PLLA was beneficial in replacing the collagen loss that occurs with box acne scarring. But this paper used a 25‐gauge sterile needle or the fanning technique. Because the experiment included box scars and rolling scar [5]. There is no paper published using a 32G, 4‐mm sterile needle for box scars, but for hyaluronic acid, it worked [14]. Rolling scars often have a normal epidermis with a width greater than the depth, caused by the subcutaneous tissue pulling the epidermis and dermis. Many studies have reported on the effectiveness of combining subcision with hyaluronic acid (HA) or poly‐L‐lactic acid (PLLA), for acne scars. These studies have demonstrated that combination therapy had better results than subcision alone [13]. Therefore, during treatment, we used a blunt needle to perform subcutaneous separation while allowing PLLA to enter the tunnels, thereby promoting collagen fiber regeneration and leveling the scars. In addition, rolling scars can be improved by subsiding the fibrous tracts.

Common side effects of the CO_2_ fractional laser in the postoperative observation of adverse reactions include postoperative erythema and postinflammatory hyperpigmentation, with an incidence rate of 8%. Common adverse reactions associated with PLLA include redness, swelling, and bruising, which typically occur within 7 days of treatment. No adverse reactions were observed.

Previously, the most concerning adverse events for physicians using PLLA treatment were nodules and granulomas [6]. The main reasons for their occurrence include (1) the particle size and volume of the product, that is, whether it has a uniform particle size and appropriate diameter [15]; (2) the concentration of the product used during preparation; (3) whether the product is evenly distributed during preparation [16]; (4) whether the patient has any immune system disorders [17]; and (5) PLLA may be an oleophilic material; therefore, after massage, it may accumulate in adipose masses, facilitating the formation of nodules [18].

In this study, no nodules were formed, primarily due to the following reasons: (1) The microspheres used had a uniform diameter ranging from 20 to 50 μm [6], and (2) the official recommendation for the concentration of polylactic acid is 150 mg/3–5 mL [6]. To ensure safety, our preparation method involved mixing 340 mg of lyophilized powder (containing 150 mg of polylactic acid) and 4–5 mL of solution. (3) Owing to the reduced hydroxymethyl cellulose content and increased mannitol content in this product, it mixes quickly, usually within 3 min, forming a monodispersed microsphere suspension system. However, during injection, the nurses were instructed to keep the product evenly shaken to ensure a uniform concentration. (4) This study excluded patients with immune system disorders and scar constitution before enrollment. However, notably, there are case reports of delayed granulomas occurring up to 70 months after treatment [19]. (5) We did not perform any massage to reduce the incidence of nodules; other adverse effects mainly included erythema (33.3%) and hyperpigmentation (6.7%) after fractional laser treatment and erythema, bruising, and pain (NRS, 4–7) after injections.

**FIGURE 8:**
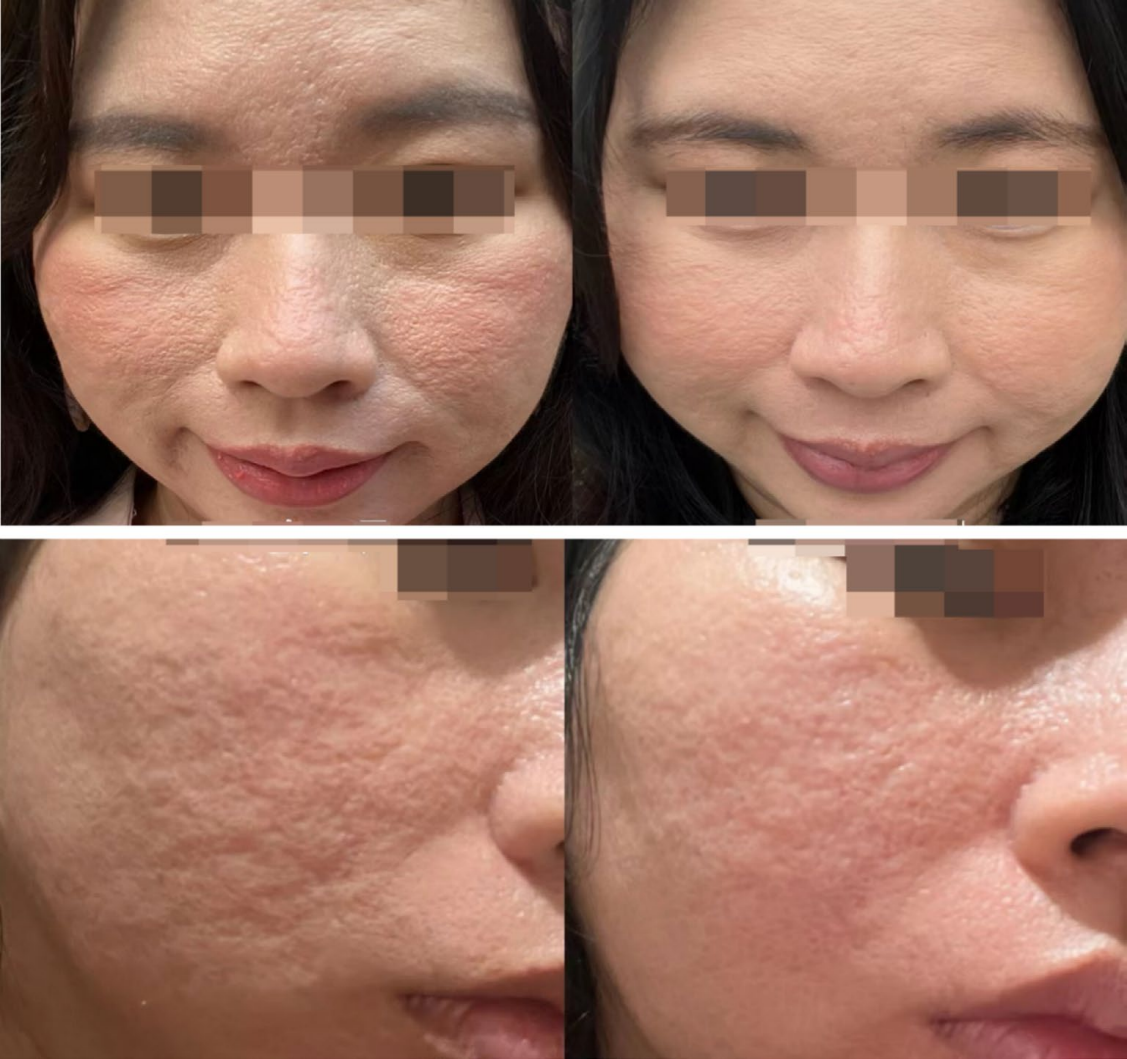
The skin texture before and after treatment with poly‐L‐lactic acid combined with carbon dioxide fractional laser, July 2022 on the left and February 2024 on the right.

In a long‐term patient who was not enrolled in this study, we also observed an improvement in skin texture (Figure 8). This patient had previously undergone 10 fractional laser treatments, resulting in a leather‐like change in skin texture [20], which became tough and resilient. After three sessions of PLLA treatment, the acne scars improved, and the skin texture showed noticeable improvement during the follow‐up visit at the end of this study. A possible reason for this is that PLLA stimulates the growth of both Type I and III collagen, with a predominance of Type III [21].

This is the first prospective clinical study in China to investigate the treatment of acne scars using this new PLLA, providing significant guidance for its clinical application. However, notably, our study has some limitations. The current study had an observation period of only 6 months, and long‐term observation may be necessary to fully assess safety. In addition, the sample size was limited.

In conclusion, the combination of CO2 fractional laser and PLLA is a safe and effective treatment for acne scars, particularly rolling and boxcar scars.

## Author Contributions

Chenxi Zhou and Weiliang Chen designed and performed the main study experiments, and wrote the manuscript. Liyuan Zhang and Luo Xiao analyzed the data. Erting Zhang and Lin Zhu collected patients' information. Xiaolei Qin and Kai Li conceived and supervised the research.

## Conflicts of Interest

The authors declare no conflicts of interest.

## Data Availability

The data that support the findings of this study are available on request from the corresponding author. The data are not publicly available due to privacy or ethical restrictions.
